# News and Notes

**Published:** 1915-01

**Authors:** 


### NEWS AND NOTES

### THIRD REGULAR CLINICAL MEETING OF GEORGIA SURGEONS' CLUB.

Atlanta, Georgia, February 25th and 26th, 1915.
Headquarters, Hotel Ansley.

OFFICERS—E. C. Davis, M. D., Atlanta, President; T. J. McArthur, M. D., Cordele, Vice President; R. M. Harbin, M. D., Rome, Secretary and Treasurer.

EXECUTIVE COMMITTEE—W. S. Goldsmith, M. D., Atlanta; G. R. White, M. D., Savannah; W. W. Battey, Jr., M. D., Augusta.

COMMITTEE ON ARRANGEMENTS—Willis Jones, M. D., Atlanta, Chairman; E. G. Ballenger, M. D., Atlanta; S. T. Barnett, M. D., Atlanta; Michael Hoke, M. D., Atlanta;

F. P. Calhoun, M. D., Atlanta; W. S. Goldsmith, M. D., Atlanta.

THURSDAY, FEBRUARY 25.

9 A. M.—Grady Hospital—Surgical Clinic—Dr. J. N. Ellis.

9 A. M.—Davis-Fischer Sanatorium—Surgical Clinic—Dr. L. C. Fischer.

10 A. M.—Atlanta Medical College—Surgical Clinic—Dr. J. L. Campbell.

11 A. M.—Wesley Memorial Hospital—Surgical Clinic—Dr. E. G. Jones.

11 A. M.—Atlanta Medical College—Genito-Urinary Clinic—Dr. W. B. Emery.

12 M.—Office 315 Grant Building—Irrigation and Drainage of Seminal Ducts and Vesicles through Vas Deferens—Dr. W. L. Champion.

12 M.—Atlanta Medical College—Genito-Urinary Clinic—Dr. A. L. Fowler.

2 P. M.—Georgia Baptist Hospital—Gynecological Clinic—Dr. M. F. Benson.

2 P. M.—Grady Hospital—Gynecological Clinic—Dr. S. T. Barnett.

2 P. M.—Piedmont Sanatorium—Surgical Clinic—Dr. F. W. McRae.

4 P. M.—Grady Hospital—Orthopedic Clinic—Drs. Hoke and Hodgson.

SURGICAL SPECIALTIES.

2 P. M.—Wesley Memorial Hospital—Eye, Ear, Nose and Throat Clinic—Dr. H. M. Lokey.

3 P. M.—Grady Hospital—Eye, Ear, Nose and Throat Clinic—Dr. F. P. Calhoun.

THURSDAY EVENING, FEBRUARY 25.

7:30 P. M.—Subscription Dinner, Hotel Ansley Banquet Hall.

Seats for the dinner and Symposium will be guaranteed by the management only to those filing their names in advance with the Hotel Clerk. (Past experience has proven the necessity of this rule.)

Short Business Meeting.

SYMPOSIUM—"SURGICAL INDIGESTION."

Banquet Hall, 9 P. M.

Definition—Dr. E. G. Jones, Atlanta.

Etiology—Stomach and Small Intestines—Dr. W. B. Hardman, Commerce, Ga.

Colon and Appendix—Dr. J. D. Chason, Bainbridge, Ga.

Gall Bladder and Ducts—Dr. A. J. Mooney, Statesboro.

Pathology: Stomach and Small Intestines, Dr. W. W. Battey, Augusta, Ga.

Colon and Appendix—Dr. F. W. McRae, Atlanta, Ga.

Gall Bladder and Ducts—Dr. H. S. Munroe, Columbus.

Symptomatology: Stomach and Small Intestines, Dr. R. M. Harbin, Rome, Ga.

Colon and Appendix—Dr. T. J. McArthur, Cordele, Ga.

Gall Bladder and Ducts—Dr. R. P. Glenn, Athens, Ga.

Diagnosis: Stomach and Small Intestines, Dr. H. J. Williams, Macon, Ga.

Colon and Appendix—Dr. G. H. Noble, Atlanta, Ga.

Gall Bladder and Ducts—Dr. W. P. Nicholson, Atlanta.

Demonstration and Radiographs—Dr. J. S. Derr, Atlanta.

Treatment: Stomach and Small Intestines, Dr. W. F. Westmoreland, Atlanta, Ga.

Colon and Appendix—Dr. W. S. Goldsmith, Atlanta, Ga.

Gall Bladder and Ducts—Dr. C. C. Harrold, Macon, Ga.

FRIDAY, FEBRUARY 26.

9 A. M.—Grady Hospital—Surgical Clinic—Dr. Willis Jones.

10 A. M.—Grady Hospital—Surgical Clinic—Dr. W. S. Goldsmith.

11 A. M.—Atlanta Medical College—Surgical Clinic—Dr. W. F. Westmoreland.

12 M.—Grady Hospital—Surgical Clinic—Dr. F. K. Boland.

12 M.—Atlanta Medical College—Dermatological Clinic—Dr. M. B. Hutchins.

12 M.—Office Candler Building—Cystoscopic Demonstration —Drs. Boyd and Shallenberger.

2 P. M.—Grady Hospital—Gynecological Clinic—Dr. G. H. Noble.

2 P. M.—Wesley Memorial Hospital—Genito-Urinary Clinic —Dr. E. G. Ballenger.

2 P. M.—Georgia Baptist Hospital—Gynecological Clinic— Dr. R. R. Kime.

3 P. M.—Atlanta Medical College—Gynecological Clinic— Drs. E. C. Davis and O. H. Matthew.

3 P. M.—Federal Prison—Cystoscopic and Ureteral Catheterization—Dr. J. Calvin Weaver.

4 P. M.—St. Joseph's Infirmary—Surgical Clinic—Dr. W. P. Nicholson.

SURGICAL SPECIALTIES.

12 M.—Office Candler Building—Demonstration to check and reduce the progress of Acquired Myopia—Dr. A. G. Hobbs.

2 P. M.—St. Joseph's Infirmary—Eye, Ear, Nose and Throat Clinic—Dr. Dunbar Roy.

3 P. M.—Grady Hospital—Eye, Ear, Nose and Throat Clinic —Dr. R. B. Ridley, Jr.

---

### THE AMBULANCE CONSTRUCTION COMMISSION.

This is the first great war in which field motor-ambulances have been extensively used. It was inevitable that many defects should be found in existing types, and in various quarters experts began to ask whether something could not be done to standardize the patterns and to improve the type. At the instance of Mr. Henry S. Wellcome, the founder of the Wellcome Bureau of Scientific Research, a Commission has been formed, and the names of members show at once that the matter is regarded as of first importance by those most intimately connected with the welfare of the wounded soldier.

Sir Frederick Treves, whose long experience and distinguished service specially fit him for the task, has consented to be the Chairman. The Admiralty is represented by the Director-General of the Medical Department, R. N., while the

Quarter-Master-General to the Forces and the Acting Director-General, Army Medical Service, represent the War Office. The British Red Cross Society is, of course, represented by Sir Frederick Treves, and the St. John Ambulance Association by Sir Claude MacDonald and Sir John Furley. The remaining members are all experts. This Commission will first and foremost act as a judging committee for the award of prizes of the value of 2,000 pounds, provided by the Wellcome Bureau of Scientific Research. These prizes are offered for the best designs of an ambulance-body which shall fit a standard pattern motor-chassis for field motor-ambulances. The last day for the receipt of competing designs is June 30, 1915. It is hoped that the competition will bring in a number of ingenious designs, from which the ideal field ambulance-body will be evolved.

It may be asked why the competition is restricted to designs for a body and not for the complete ambulance, including a chassis. The reason is that a chassis takes much longer to build than a body, and that, when war breaks out, it is impossible to get at short notice anything like a sufficient number of any one type of chassis. On the other hand, a standardized body to fit any chassis of approved dimensions can be constructed in numbers at comparatively short notice. And a perfected body is badly wanted to insure complete comfort for the wounded.

It is hoped that the information obtained by the competition, and in other ways, will be published in some permanent form, available for future reference. Probably in addition to one design of special excellence, there will be submitted various ingenious suggestions which may be incorporated in the pattern design approved by the Commission. For these, a portion of the prize-money has been set apart. The first prize is of one thousand pounds, the second of five hundred and the third of three hundred pounds. All details of conditions may be obtained from the Secretary, the Ambulance Construction Commission, 10 Henrietta Street, Cavendish Square, London, W. The competition is open to citizens of all nations.

### A MODERN SANATARIUM FOR THE TREATMENT OF NERVOUS DISEASES.

Probably the most distinctive mark of civilization today as setting it off from the barbarous habits of our continuously fighting ancestors is the care and attention we give to the sick and feeble. In the olden times as soon as one was in the way of the family or hindered the tribe in the acquisition of provender, he was killed off or left to starve by himself. Rare exceptions arose when some alien minded individual attracted awed attention by his peculiarities even though he was bodily feeble. Then he was made an object of veneration and became a misleading and dangerous guide to his fellows.

In civilized times the sick have been cared for and helped and the profession of medicine has grown through various curious stages to its present concentrated and direct activities. During this period the care of the insane showed the most picturesque condition and made the most marked changes from a wild and superstitious empiricism to an intelligent and scientific procedure. Demoniac possession as the cause of insanity long held sway, but it is a dead idea, and the sick man is no longer subjected to incantations and exorcisms nor must he suffer painful restraint of his bodily activities.

There does remain in the minds of most people a feeling of shame if any member of the family suffers with mental trouble and the thought still leads to the seclusion of such a patient from the attention of the world. The fact that this seclusion, if given in the right way, is the best thing for the patient, inasmuch as it provides for him the rest he needs, militates in some measure toward perpetuating the secretive process. It is just that which modern Sanatariums and thoughful physicians are striving to overcome.

In the first place, the teaching is everywhere made prominent that insanity in any and all its forms, is not to be looked upon as shameful any more than is pneumonia or blindness. It is a disease or an unfortunate condition that can either be cured completely or mitigated so that a great deal of comfort and happiness can be given to the sufferer. During the first decade of the century, discoveries have been made that lead to the cure of cases of insanity which were previously classed as beyond the reach of all but custodial care, and today in well-equipped institutions things are being done that would have been looked upon as miracles—not away back when the devils were cast out, but less than twenty years ago when we were only learning to do away with physical restraint in the care of disturbed people.

It is true, too, the times have brought new problems in the shape of strange drug habits and of peculiar occupations, but these are being met as soon as they arise with prompt recognition due to the advancement made all along the line in medicine and especially in toxicology both inorganic and organic. We have learned how to see things more quickly and with greater accuracy and the insane are profiting fully as much as those patients needing surgical care.

The new institutions springing up over the country for the care of the insane keep pace with those constructed for surgical purposes, though their clientele remains more confidential and the public attention is not drawn to them.

A conspicuous example of this is the Cheston King Sanitarium near Atlanta. In the first place, its location is far enough away from the busy city to give its patients that quiet and restfulness essential to recovery, while at the same time it country homes of prosperous people. It is not hidden away. There is nothing suspicious or furtive about it. Not four hundred yards from its doors runs one of the principal railroads of the South and a neat station is there for the convenience of friends desiring to visit the sick. The grounds of a beautiful Country Club adjoin it and the general atmosphere is that of attractive quiet.

With this impression in mind, one reaches the fine building, constructed along the lines of a large residence or perhaps a small and delightful hotel. It is fire-proof, but that fact does not stare one out of countenance. Entering its pleasant hallways the impression is instantaneous that here are comfort and cleanliness combined with quiet efficiency. The offices are small rooms where people can talk in confidence, not great, severe spaces that make one wonder and stare. A patient arriving there is likely to feel at once that he is in consultation with a doctor and where he can gain relief rather than in a big institution where his record is being taken as a routine measure.

The privacy is just such as one would expect in going to a doctor's office and there is nothing oppressive or forbidding about it.

The rooms in the two wings are so planned that every one has a fine outlook. There is really no "back" outlook. They are furnished neatly and comfortably in the most modern manner and everything is new. Many rooms have baths attached and all are equipped with running water and the best of sanitary plumbing. Where rooms are without baths, they are close to bath rooms opening on the same passageway. In close proximity also are amusement rooms and places of assembly if such is desired. Wherever one looks there i scheerfulness and comfort and equal opportunities for privacy or companionship as one may choose.

In one part of the house various occupations are provided as means of treatment. These, together with the indoor games, are light and pleasant and serve to give practice to the mind that is learning once more to concentrate and control its activities.

The dining rooms and large kitchen which are in a wing by themselves so that no odors from them reach the other quarters, are a pleasure to behold. In the first place, they are high from the ground and have windows everywhere. Light insures cleanliness in a place so equipped with modern apparatus that cleanliness is easy. The kitchen is so conveniently located that meals can be served hot and fresh to such patients as are confined to bed.

The laundry is ample in size for all demands upon it and has proper machinery for sterilizing and cleansing of the clothing as well as for the finishing of linen to the satisfaction of fastidious patients.

The buildings are heated by vapor and the boiler room capacity is double the need. We made a visit on the coldest day in 1914 and found the entire place perfectly comfortable when the heating apparatus was under less than half pressure.

The water supply comes from an artesian well 250 feet deep, that gives a spontaneous flow of over ten times the greatest need of the Sanitarium. This has been found of rare purity. The water is pumped everywhere throughout the institution and provides amply for the modern hydro-therapeutic installation in one of the basements.

The lighting comes from the wires of the city power and lighting plant and is constantly availible. The installation of the lamps is entirely upon the indirect principle so that there is no glare. The current is received and handled by transformers so that it can be used for therapeutic measures whenever needed.

The grounds surrounding the Sanitarium are commodious and well arranged. At the front are winding drives and a sunken garden so that the outlook from the porch is pleasing. At one side there are tennis courts and handball courts where these games can be enjoyed all the year round. It should be remembered that there is practically no snow here and that while many of the winter days are cold and bracing, they are always inviting and the outdoor life allures all but the bed-ridden. Beyond the institution there are extensive gardens where fresh vegetables are raised and where an excellent dairy is maintained. The grazing land merges into the woods at the far end of the estate over a gentle declivity. The fine cattle lending life to the vistas would inspire a Troyon to special pictures.

And these grounds are protected against disease, for they are amply and carefully drained and the drainage, together with that from the Sanitarium, is cared for by a sewage disposal plant of the latest scientific construction.

Several fine roads spread out from the Sanitarium along which one may ride through a beautiful and historic country-side. The climate invites outdoor life in all seasons because of its moderate rainfall and mild temperature. The winters are never severe and winter clothing is needed only from November to the middle of April. The summers are long and delightful because the nights are cool and the heat is never so great as it is in the southern tier of counties in New York for example.

To people living in New York or other states of similar latitude, the spring time here is a revelation, for the season is real and charming, telling its own wonderful story without the aid of imaginative and farfetched poems to describe it. It is in truth a glorious season with many days and nights in long succession such as inspired the poet to ask, "What is so rare as a day in June?" One can be certain that the question was never penned by a resident of the Piedmont region around Atlanta. The spring is filled with them.

The comparison has been hinted between this modern hospital and those for surgery and general medicine. It is extended in the provision that is made for such surgery as the treatment of mental diseases requires. It is enough to mention spinal injections when remedies of that character are indicated. Proper provision is made for this and such other surgical and mechanical treatment as may be needed.

The Sanitarium is not large. Its capacity is fifty beds and it is so built that it cannot be crowded. There are no dormitories. It is under the constant care of experienced alienists who are residents in their homes upon the grounds. Besides these there is a small and efficient staff of consultants resident in Atlanta. Nothing is done for show. Everything must have its effectiveness in the comfort or welfare of the patients or it is set aside. There is hardly time as it is to do the things that the advances in sciences have shown to be of value and to give the close personal attention without which there can be no success in the treatment of diseases of the mind.

It is needless to say that the Sanitarium is not a show place and that under all circumstances the personal privacy of its patrons is regarded as inviolable; but neither is it a forbidden land.

Any physician is welcome to inspect it completely at any time and to assure himself that no statement made above is exaggerated. Moreover, any layman having a friend or relative needing such care as the hospital provides, will be given every opportunity to satisfy himself as to the conditions under which the patient would live.

Thus we have in Atlanta not only fine general hospitals in the city proper where every medical and surgical need is met with the latest scientific advance to satisfy it, but also there is just without our walls a most excellent example of a hospital for the treatment of mental diseases.

